# Death certificate completion skills of hospital physicians in a developing country

**DOI:** 10.1186/1472-6963-13-205

**Published:** 2013-06-06

**Authors:** Ahmed Suleman Haque, Kanza Shamim, Najm Hasan Siddiqui, Muhammad Irfan, Javaid Ahmed Khan

**Affiliations:** 1 The Aga Khan University Hospital, Stadium Road, P.O. Box 3500, Karachi 74800, Pakistan

## Abstract

**Background:**

Death certificates (DC) can provide valuable health status data regarding disease incidence, prevalence and mortality in a community. It can guide local health policy and help in setting priorities. Incomplete and inaccurate DC data, on the other hand, can significantly impair the precision of a national health information database. In this study we evaluated the accuracy of death certificates at a tertiary care teaching hospital in a Karachi, Pakistan.

**Methods:**

A retrospective study conducted at Aga Khan University Hospital, Karachi, Pakistan for a period of six months. Medical records and death certificates of all patients who died under adult medical service were studied. The demographic characteristics, administrative details, co-morbidities and cause of death from death certificates were collected using an approved standardized form. Accuracy of this information was validated using their medical records. Errors in the death certificates were classified into six categories, from 0 to 5 according to increasing severity; a grade 0 was assigned if no errors were identified, and 5, if an incorrect cause of death was attributed or placed in an improper sequence.

**Results:**

223 deaths occurred during the study period. 9 certificates were not accessible and 12 patients had incomplete medical records. 202 certificates were finally analyzed. Most frequent errors pertaining to patients’ demographics (92%) and cause/s of death (87%) were identified. 156 (77%) certificates had 3 or more errors and 124 (62%) certificates had a combination of errors that significantly changed the death certificate interpretation. Only 1% certificates were error free.

**Conclusion:**

A very high rate of errors was identified in death certificates completed at our academic institution. There is a pressing need for appropriate intervention/s to resolve this important issue.

## Background

Death certificates are an important tool to ascertain population based mortality and other vital statistic. The amount of data contained in each death certificate is limited, but essentially includes identification/demographic data, date and location of death, morbidity data and the cause of death. These certificates may play a role in medico-legal investigations, declaration of health events in public health researches [1-3], and epidemiological studies to evaluate mortality in a community. These certificates can also be a valuable source of census studies [4-6]. They can also be helpful for families to understand the course of death and become cognizant of the inherited risk factors for certain disease.

Death certificate data is used to calculate vital statistics and inaccuracies lead to errors in population based studies that rely on these statistics. Literature has shown that the error rates in death certificate completion are still very high, ranging from 25% to 78% in hospital-based studies [7-13], and from 16% to 56% in population-based studies [14-16]. These errors can range from simple omissions to illegible hand writing and use of abbreviation, to inaccurate causes and manners of death. A vast majority of these studies focus solely on the presence or absence of specific disease [17,18] entities thus allowing an assessment of degree of misclassification in death certification. However, only a few studies have attempted to classify all possible errors into categories [19,20] in an effort to identify the common ones and separate them into minor and major inaccuracies, which would allow for appropriate measures in teaching/ training in an effort to reduce their future occurrence.

In Pakistan, doctors usually do not receive sufficient training on the death certificate completion skills. This results in inaccurate death certificates, thereby compromising the effectiveness of the health information in the national data-base. Accurate information drawn from cause of death statements on death certificate is crucial for effective planning and evaluation of health care programs and health status.

This study was aimed at determining the accuracy of death certificates, to identify the types and frequency of errors and to press the need to improve the death certificates writing skills of the physicians.

## Methods

This retrospective study was aimed to examine and analyze the accuracy of death certificates for patients who died under the general medicine service during a 6-month period (July 2009 to December 2009) at The Aga Khan University Hospital (AKUH), Karachi, Pakistan. Our hospital is a 560 bedded tertiary care teaching hospital located in the metropolis city of Karachi, which is the second most populated city in the world and accommodates approximately 16 million multiethnic inhabitants. The death certificate used to report the cause of death at our institution is in accordance with the World Health Organization (WHO) guidelines. The institutional Ethics Committee reviewed and approved the research protocol.

The following information was obtained from the death certificates:

(i) Demographic characteristics (e.g. age, sex, marital status, residential address, date of birth and date of death) of the deceased patients.

(ii) Administrative details, including date of admission, place and time of death, who completed the death certificate, and whether autopsy had been performed or not. The signature and identification profile of the certifier were also verified.

(iii) Medical data indicating the immediate underlying cause of death and co morbidities.

The information abstracted from individual death certificates was collected using an approved standardized form. Accuracy of this information was then validated and corroborated by undertaking a review of the relevant information from medical records. This initial evaluation was conducted by two trained reviewers and in case of discrepancies a third investigator made an independent judgment before a final agreement was reached through consensus.

We used a predetermined error grading scale (Table 1) to assess the accuracy and thoroughness of individual death certificates. Errors were assigned a grade from zero to V with increasing severity. A grade zero was assigned if no error was identified. Error grade IA included incomplete/inaccurate demographics and 1B an inability to confirm (from the medical records) that the signatory had attended the patient prior to death. Error grade II or III included missed or complete omission of the documented comorbid conditions respectively. Grade IV errors included an inappropriate immediate cause of death (i.e. the final disease or condition resulting in death) or only mentioning a mechanism(s) of death (or mode of dying). Grade V errors included incorrect underlying cause(s) of death (i.e. the disease or injury that initiated the train of morbid events resulting in death) or stated in an improper sequence. Grade IV and V errors were thought to significantly change the death certificate interpretation.

**Table 1:** Grading scale used to assess death certificate errors

**Grade 0**	No errors
**Grade IA**	incomplete/inaccurate demographics
**Grade IB**	Whether the signatory attended the patient could not be confirmed
**Grade II**	Co-morbidities list incomplete
**Grade III**	Co-morbidities not listed
**Grade IV**	Inappropriate immediate cause of death or only a mechanism(s) of death (or mode of dying) given *
**Grade V**	Underlying cause(s) of death was incorrectly attributed or placed in an improper sequence

*Since multiple diseases can cause death by the same mechanism, mechanism of death usually provides little useful information [21].

The study was reviewed and approved by the departmental ethical committee.

### Statistical analysis

The errors identified in the death certificates were categorized into six grades (Grade 0 to V) according to increasing severity (as described in the method section). These grades were analyzed and presented as number and percentages. For categorical variables frequency and proportions were calculated. Proportions were compared using chi square test and means were compared using t-test. Statistical analysis was conducted using Statistical Package for Social Sciences (SPSS, version 19). A p-value of <0.05 was considered to indicate statistical significance.

## Results

A total of 223 deaths occurred during the 6 month audit period. 9 certificates were not accessible and 12 patients did not have complete medical records available. 202 certificates were therefore included in the final analysis. Only 2 (1%) Death certificates had no errors. The most frequent errors in the ascertainable data included Grade IA errors (Table 2) involving patients’ demographics and Grade V errors regarding cause/s of death (Table 2). At least one error was found in 185 (92%) certificates regarding either patient’s name, age, date of birth or missed consultant name. 156 (77%) certificates had 3 or more errors of varying grades (Table 3). 124 (62%) certificates had a combination of most serious grade IV and V errors. The rate of inappropriate certification however did not differ significantly with age or gender of the deceased.

**Table 2:** Number and percent of errors in 202 death certificate by different grading*

**Errors**	**Number**	**Percent of cases**
Grade 0	2	1%
Grade IA	185	92%
Grade IB	100	49%
Grade II	37	18%
Grade III	66	33%
Grade IV	125	62%
Grade V	176	87%

*Refer to Table 1.

**Table 3:** Distribution death certificates error/s by number

**Number of errors**	**Number of death certificate**
One error	11
Two errors	33
Three errors	56
Four errors	66
Five errors	34
Total	200

## Discussion

Death certificate has been used as a health indicator and as a monitoring tool for public health policy. They enable us to describe patterns within a whole population. Moreover, the absence of reliable data on causes of death impedes the structuring of health-related activities and can thus result in misleading appraisals of research and improper decisions regarding health care.

Most physicians confuse the cause of death with the mechanism of death [21]. The cause of death is a distinct entity, and is etiologically specific. Examples include subarachnoid hemorrhage, COPD, and myocardial infarction. The mechanism of death, on the other hand should contain information on all other diseases, conditions, or injuries that lead to the physiologic derangement or a biochemical disturbance that eventually contributed to the cause of death. Examples include various arrhythmias, cardiopulmonary failure, renal failure, hypovolemic shock, and sepsis. One reason for this confusion may be that medical therapy is often aimed at modifying or ameliorating mechanisms rather than causes, thereby focusing attention on the former to the disregard of the latter [21].

Due to their lack of etiologic specificity, mechanisms or mode of death should not appear on death certificates [21-25]. Nevertheless, in daily clinical practice, a definite cause of death is not always identified. In our study, 62% cases were labeled with mechanism of death rather than cause of death. Hanzlick proposed some principles for including or excluding mechanisms of death when writing the cause-of-death statement [24]. However, a recent evaluation of WHO's web based training tool for coders and certifiers showed that despite training, further improvement particularly in the areas of reporting of the correct and complete sequences from underlying cause through intervening causes to the immediate cause of death was still required [26]. Similar outcomes have been reported in the past across the globe. In 1993 Jordan and Bass [11] showed that 31.9% of a sample of death certificates completed at a Canadian tertiary care teaching hospital contained such errors. El-Nour et al. found 45% of the death certificates contained such errors [27] while a national study in Taiwan revealed 7% of such errors [13].

The accuracy of the death certificate could be audited and confirmed from a complete medical record. The best certifier should be the treating physician of the deceased who recorded all details of his/her condition [28] on the medical record so as to put them in proper sequence in the death certificate. Most of the doctors do not refer to the corresponding diagnoses in the medical record to identify the underlying cause of death, the antecedent cause(s) and the direct cause of death. According to the study of Lu et al. [13], in most death certificates, the certifying physician copied the admission or discharge diagnoses directly to the cause-of-death section on the death certificate. If the certifying physician copies the admission or discharge diagnoses directly to the cause-of-death section on the death certificate, there will be many diagnoses listed without causal relationships. Take, for example, a patient admitted to the hospital mainly for complications of diabetes, but who also had emphysema. The first admission diagnosis would be diabetes and the second would be emphysema, according to the severity of the problems. If the certifying physician entered diabetes on the first line of the death certificate and emphysema on the second, the certificate would have a Grade V error because it would suggest that emphysema was the cause of diabetes.

These error issues are not limited to the developing countries. More than 50% of general practitioners in the United Kingdom and in the US reported being insufficiently instructed about the process of death certification [29,30]; many said that their first contact with a death certificate occurred when they first managed a death event [31].

These inaccuracies stem from a lack of knowledge among doctors on how to identify and select the underlying cause, direct cause and antecedent cause(s) of death [24]. Another plausible explanation contributing to these mistakes is the length of illness that leads to death. If a person dies after a long, well-characterized illness, the cause of death on the certificate is likely to be more accurate than a sudden or unobserved death. This lack of adequate information about the decedent’s disease history produces a more narrowly characterized cause of death, increasing the likelihood of important omissions and eventually impacting disease statistics [31].

Direct comparison of our study with previous studies is difficult due to differences in the definitions and interpretations of error between studies. However, there is uniform agreement among most of these studies, including ours that the wrong cause or manner of death and a lack of an acceptable underlying cause of death qualify as major errors. In our study, 124 (62%) certificates had a combination of such errors that significantly changed the death certificate interpretation and would therefore have major public health implications. With regards to demographic data, it is usually assumed to be accurate and not subject to significant error. However, one of the studies found some errors in recording place of residence on death certificates [32]. In our study, 92% of the death certificates had errors associated with the inaccurate or incomplete demographics. Similarly we found that approximately in half of the death certificates (49%) it was not possible to confirm that the certifier had actually seen the patient.

Our study has some major limitations. Firstly, it’s retrospective design. Secondly, in Pakistan and many other developing countries, there are religious, cultural or traditional taboos around necropsy and it is usually refused. Therefore generating a cause of death solely from review of medical records, without the direct involvement of the attending physician/team can result in some degree of misinterpretation despite employing multiple reviewers [33]. Thirdly, this was a single institution practice assessment and may not be generalizable to other establishments.

In conclusion, we found substantial shortcomings in the death certification practices locally. There is a pressing need for appropriate intervention/s to improve and enhance the accuracy of physicians’ death certificate completion skills.

## Competing interests

All authors declare that they have no competing interests.

## Authors’ contributions

The proposal was prepared by JAK in consultation with ASH and MI. ASH, JAK, KS and NHS have analyzed and interpreted the patient data. ASH, NJM, MI and KS were major contributors in writing the manuscript. All authors read and approved the final manuscript.

## Pre-publication history

The pre-publication history for this paper can be accessed here:

http://www.biomedcentral.com/1472-6963/13/205/prepub
